# Facilitating functional annotation of chicken microarray data

**DOI:** 10.1186/1471-2105-10-S11-S2

**Published:** 2009-10-08

**Authors:** Teresia J Buza, Ranjit Kumar, Cathy R Gresham, Shane C Burgess, Fiona M McCarthy

**Affiliations:** 1 Department of Basic Sciences, College of Veterinary Medicine, Mississippi State University, Mississippi State, MS 39762, USA; 2 Institute for Digital Biology, Mississippi State University, Mississippi State, MS 39762, USA; 3 Life Sciences and Biotechnology Institute, Mississippi State University, Mississippi State, MS 39762, USA; 4 Mississippi Agricultural and Forestry Experiment Station, Mississippi State University, Mississippi State, MS 39762, USA; 5 Department of Computer Science and Engineering, Bagley College of Engineering, Mississippi State University, Mississippi State, MS 39762, USA

## Abstract

**Background:**

Modeling results from chicken microarray studies is challenging for researchers due to little functional annotation associated with these arrays. The Affymetrix GenChip chicken genome array, one of the biggest arrays that serve as a key research tool for the study of chicken functional genomics, is among the few arrays that link gene products to Gene Ontology (GO). However the GO annotation data presented by Affymetrix is incomplete, for example, they do not show references linked to manually annotated functions. In addition, there is no tool that facilitates microarray researchers to directly retrieve functional annotations for their datasets from the annotated arrays. This costs researchers amount of time in searching multiple GO databases for functional information.

**Results:**

We have improved the breadth of functional annotations of the gene products associated with probesets on the Affymetrix chicken genome array by 45% and the quality of annotation by 14%. We have also identified the most significant diseases and disorders, different types of genes, and known drug targets represented on Affymetrix chicken genome array. To facilitate functional annotation of other arrays and microarray experimental datasets we developed an Array GO Mapper (*AGOM*) tool to help researchers to quickly retrieve corresponding functional information for their dataset.

**Conclusion:**

Results from this study will directly facilitate annotation of other chicken arrays and microarray experimental datasets. Researchers will be able to quickly model their microarray dataset into more reliable biological functional information by using *AGOM *tool. The disease, disorders, gene types and drug targets revealed in the study will allow researchers to learn more about how genes function in complex biological systems and may lead to new drug discovery and development of therapies. The GO annotation data generated will be available for public use via AgBase website and will be updated on regular basis.

## Background

The development of microarray high-throughput screening platforms for chicken is an important step for gene expression profiling in changes occurring in avian as a response to different challenges and stimuli [1-3]. The chicken research community uses microarrays for a wide range of applications, including gene expression analysis [1,4], exon expression analysis [5-7], novel transcript discovery [8], genotyping [9,10] and resequencing [11,12]. In addition, microarray analysis can also be combined with chromatin immunoprecipitation to perform genome-wide identification of transcription factors and their respective binding sites [13].

According to statistics obtained from "Gallus Expression *in Situ *Hybridization Analysis" (GEISHA; http://geisha.arizona.edu/geisha/microarray.jsp; 03/14/2009), there is already significant resources constructed for the "Whole Genome" Chicken Microarrays. Listed in GEISHA are: 1) Arizona *Gallus gallus *20.7 K Long Oligo Array, 2) Affymetrix array which cover 32,773 transcripts corresponding to over 28,000 chicken genes, 3) FHCRC Chicken 13 K Array, 4) University of Delaware-Larry Cogburn which produced UD_Liver_3.2 K, UD 7.4 K Metabolic/Somatic Systems, Chicken Neuroendocrine System 5 K and the DEL-MAR 14 K Integrated Systems and 5) ARK Genomics which offers a 1,153 clone chicken embryo array, a 5,000 cDNA chicken immune array, and a 4,800 clone chicken neuroendocrine array. Gene Expression Omnibus (GEO), publicly accessible through the World Wide Web at http://www.ncbi.nlm.nih.gov/geo, is a curated public repository for high-throughput gene expression data [14,15]. Platform is one of central data entities of GEO which contains a list of probes that define what set of molecules may be detected and can easily be browsed, queried and retrieved to fit user's interests [14,16].

Comprehensive annotation of these arrays will benefit chicken researchers, because they will be able to functionally model their expressed dataset to obtain relevant information about their biological system. However, most arrays are not associated to any functional information. The only array that is comprehensively annotated to GO is the Affymetrix chicken GeneChip array [17]. This array is the mostly used for gene expression studies as shown in a survey when the chicken research community was polled in July 2008 http://doodle.com/participation.html?pollId=zwvmhpt5t23tvfv8). The Affymetrix NetAffx database links probesets on Affymetrix GenChip microarrays to GO using data from the GO Consortium [18]. However, the GO evidence codes are not linked to any reference that was used to make functional assertions. This is a challenge to researchers who want to associate their dataset with functional information at the same time showing supporting evidence. For example, use of an experimental evidence code in a GO annotation should be associated with a paper that displays results from a physical characterization of a gene/gene product being annotated. This allows the researcher to access the detailed information that was used to make the GO annotation.

In this study we have re-annotated all gene products associated with probesets on Affymetrix chicken genome array using GO standards. However, the GO describes normal gene or gene product function [19] such that information about which genes are associated with significant diseases and disorders and which are known to be drug targets is not captured using the GO. This type of information would clearly benefit researchers in modeling diseases. We therefore used Ingenuity Pathway Analysis to identify significant diseases, disorders, drug targets and types of gene represented on Affymetrix chicken genome array. Furthermore, we demonstrate how other microarrays can be annotated using the annotations from Affymetrix chicken array.

## Results

### Initial assessment of structural and functional annotation of chicken array

Most of chicken arrays currently available are linked to either gene or gene products but very few of the arrays are annotated to any functional information (Table 1). The Affymetrix chicken array was chosen for this study because it represents most of genomic elements annotated on chicken genome. Initial assessment of annotation of Affymetrix chicken genome array are shown (Additional file 1). Over 97% of chicken Affymetrix probesets are mapped to 27,852 genes or gene products in total. Other probesets represented on this array are for studying 17 different avian viruses. About 51% of the probesets are associated with GO annotations made for 12,457 genes or gene product.

**Table 1:** Initial assessment of structural and functional annotation of chicken array

		Cross reference			
Name of Microarray	Size			GO	Evd*
		Gene/EST	Protein		
ARK-Genomics G. gallus 20 K v1.0 (GPL5480)	22,176	**+**	**-**	**-**	**-**
ARK-Genomics G. gallus 13 K v4.0 (GPL5673)	27,648	**+**	**-**	**-**	**-**
Affymetrix GenChip^® ^chicken genome array	38,535	**+**	**+**	**+**	**+**
Chicken 44 K custom Agilent microarray (GPL4993)	42,034	**+**	**+**	**-**	**-**
Arizona Gallus gallus 20.7 K Oligo Array v1.0 (GPL6049)	21,120	**+**	**-**	**-**	**-**
FHCRC Chicken 13 K Array (GPL1836)	15,769	**+**	**-**	**-**	**-**
Custom 4 × 2 K miRNA microarray (#4166) (GPL7472)	1,412	**+**	**-**	**-**	**-**
Chick Pineal 2004 (GPL1289)	9,056	**+**	**+**	**-**	**-**
DEL-MAR 14 K Integrated Systems(GPL1731)	19,200	**+**	**-**	**-**	**-**
Avian Innate Immunity Microarray (AIIM) (GPL1461)	14,877	**+**	**-**	**-**	**-**
UD 7.4 K Metabolic/Somatic Systems (GPL1737)	7,680	**+**	**-**	**-**	**-**
UD_Liver_3.2 K (GPL1742)	3,456	**+**	**-**	**-**	**-**
Chicken_Neuroendocrine_System_5 K (GPL1744)	7,000	**+**	**-**	**-**	**-**

Different chicken arrays (column 1) have different gene products represented on them (column 2). Column 3 & 4 shows whether the printed transcripts are linked to a gene (G), mRNA (R), EST or protein. GO in column 5 indicates GO functional annotation linked to gene products represented on these arrays and evd (column 6) indicates evidence code supporting the functional information. The (+) or (-) in columns 3 – 6 indicates presence or absence of the parameter in that specific column.

**+ **or **- **shows that the array is linked or not linked.

*Evidence that support the GO annotation

### Functional annotation and GO annotation quality

**Figure 1:**
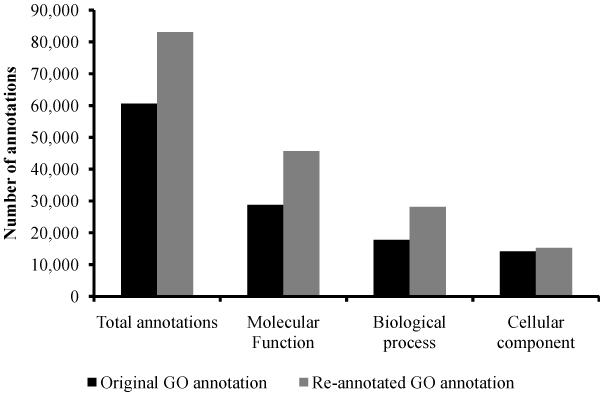
**Functional annotation of Affymetrix chicken genome array**. Original annotation of Affymetrix chicken array (grey bars) were compared with re-annotated GO (black bars). All biological ontologies show improvements realized from the re-annotation.

**Figure 2:**
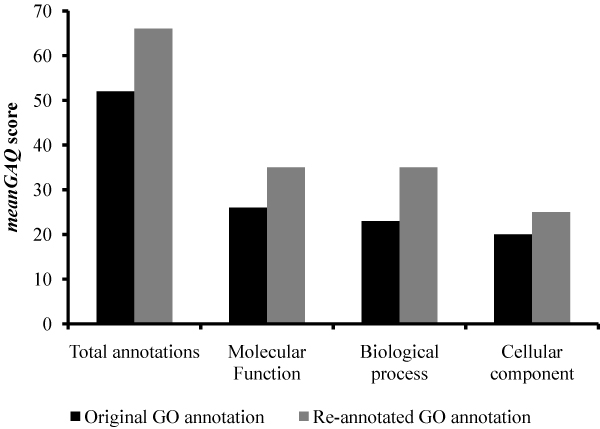
**The mean GAQ score of the GO annotation**. The mean GAQ scores are calculated for both original (black bar) and re-annotated (grey bar) GO annotations. The mean GAQ score is based only on the unique gene products with GO, not individual the probesets.

The GO annotation of Affymetrix chicken probesets does not show any reference supporting the evidence of the annotation as pointed out in methods section. We re-annotated all gene products represented on this array, regardless of their initial annotations, according to GO standards. We were able to increase the number of GO annotations in all three ontologies (Figure 1); re-annotation increased the total GO annotations by 45%, the number of annotated gene products by 10% and the number of probe sets linked to annotated gene products by 13%. Moreover, the quality of the original GO annotations in all three GO ontologies, as determined by GAQ score [20], was improved by the additional annotations (Figure 2). Briefly, the GAQ score quantitatively assess the level of detail provided by the GO annotation and the type of evidence used to make the annotations. The overall mean GAQ score of all annotations regardless of biological ontology, increased from 52 to 66.

**Figure 3:**
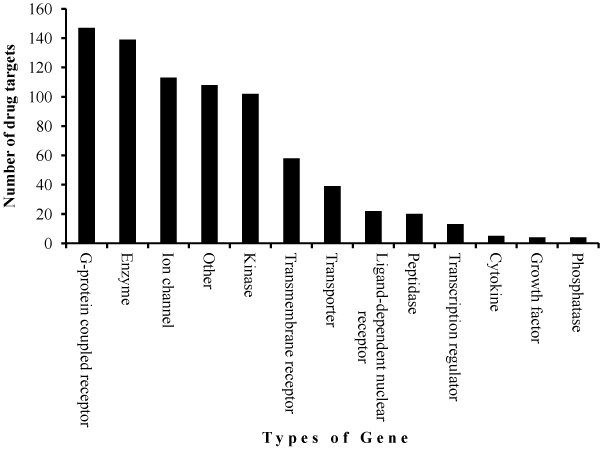
**Types of genes and drug targets represented on Affymetrix GenChip^® ^chicken genome array Sample figure title**. The probesets matching different types of genes (A) were determined by using Ingenuity Pathway Analysis software. Some probesets were mapped to genes that are considered drug targets (B).

Additional functional information was obtained using the Ingenuity Pathway Analysis (IPA) tool to identify the significant biological functions, diseases and disorders that are represented on Affymetrix chicken genome array (Table 2). The most significant diseases and disorders represented on this array are cancer and genetic disorders, respectively. Cell death was identified to be the most significant molecular and cellular function while organismal survival was the most significant process among the physiological system development. Different types of genes and known drug targets were also identified (Figure 3).

**Table 2:** Biological functions represented on Affymetrix chicken GenChip^® ^array

Biological Function	Number of Genes	P-value*
**Diseases and Disorders**		
Cancer	2,298	2.43E-53 – 6.86E-08
Neurologic disease	1,219	4.94E-52 – 6.76E-08
Genetic disorder	1,152	6.69E-37 – 6.69E-37
Cardiovascular disease	583	3.18E-36 – 6.17E-08
Developmental disease	554	6.01E-30 – 6.17E-08
**Molecular and Cellular Functions**		
Cell death	1,604	1.19E-55 – 6.51E-08
Cellular growth and proliferation	1,774	6.66E-42 – 4.87E-08
Cellular development	1,231	1.00E-35 – 5.68E-08
Gene expression	1,231	2.82E-35 – 2.21E-08
Cellular movement	931	1.89E-32 – 6.78E-08
**Physiological System Development and Function**		
Organismal survival	718	5.95E-38 – 1.18E-12
Tissue development	920	6.07E-36 – 4.30E-08
Organismal development	879	5.40E-34 – 5.36E-08
Organ development	585	2.33E-33 – 4.90E-08
Tissue morphology	666	2.03E-27 – 1.20E-08

Significant biological functions represented on Affymetrix chicken genome array.

*Based on Fisher's Exact Test P-value ≤ 0.05

### Tool for array GO mapping

**Figure 4:**
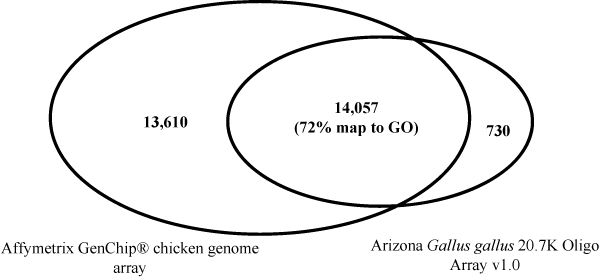
**Distribution of genes and gene products represented on Affymetrix and Arizona chicken array**.

Improved functional annotation of Affymetrix chicken array proved to facilitate the annotation of other arrays, such as the Arizona Gallus gallus 20.7 K Oligo Array v1.0 (GPL6049). An Array GO Mapper (*AGOM*) tool developed in this study was able to map Entrez genes, Ensembl genes and GenBank accessions from the Arizona array to Affymetrix annotations in order to retrieve GO annotations. We successfully identified 79% of genes that were common in both arrays (Figure 3), out of which 72% were mapped to GO annotations (Figure 4). The total number of GO annotations generated for Arizona array was 60,846. An example of output generated by *AGOM *is shown on additional file 2 which includes only the first 1,000 gene association lines generated for Arizona chicken array. The mean GAQ score associated with the GO annotations retrieved was 59 and was calculated by summing up all GAQ scores of all 60,846 GO associations and dividing these by the number of annotated gene products. These results provide an initial assessment of GO annotations available for the Arizona chicken array and demonstrates how GO annotations can be transferred to identical transcriptional elements represented on multiple arrays.

## Discussion

The major challenge that faces microarray researchers is interpretation of hundreds of differentially expressed genes into a biologically relevant context. The Gene Ontology (GO) Consortium provides a controlled vocabulary to annotate the biological knowledge associated with genes or gene products. In order to make the functional interpretation of microarray dataset less challenging, microarray developers can associate their arrays with functional information.

However, most chicken arrays either have no associated GO information or do not follow the GO annotation standards [21]. In this study we have re-annotated and improved the GO annotation of Affymetrix chicken genome array to facilitate annotation of other chicken arrays and microarray experimental datasets. Further, we developed the Array GO Mapper (*AGOM*) tool to generate GO annotations for chicken arrays with no GO information or for microarray experimental datasets and demonstrated its utility by annotating the Arizona chicken array which had no associated GO information. By implementing *AGOM *researchers will not only obtain functional information for their experimental dataset but will also obtain GAQ scores associated with each GO term retrieved. This will help researchers determine the quality of annotations made to their datasets and also help tracking the improvement made by any additional GO when there are any updates.

We also provided additional functional information not covered by the GO but is associated with the Affymetrix chicken genome array. This additional data broadens the ability of array users to model their datasets, for example infectious disease datasets. The additional information obtained on diseases, disorders and known drug targets represented on this array will provide light to future research in drug and therapy development.

## Conclusion

Improved amount and quality of GO annotations of gene products represented on the Affymetrix chicken genome array will help researchers to model their genes of interest to high quality functional information by using AGOM tool. The existing chicken microarray studies can use AGOM and this demonstrates how this tool can enhance functional annotation in these studies. Annotation of microarrays of other species will be included in the future. The top significant diseases and disorders represented on the chicken array correlate well with how the chicken is used as a biomedical model organism to study human diseases and development. The identified gene types and drug targets allows researchers to learn more about how genes function in complex biological systems and may lead to new drug discovery and development of therapies.

## Methods

### Initial assessment of structural and functional annotation of chicken array

We downloaded 12 chicken array platforms deposited in the NCBI Gene Expression Omnibus (GEO: http://www.ncbi.nlm.nih.gov/geo/) database (Table 1). Affymetrix GenChip chicken genome array annotations were downloaded from the Affymetrix website http://www.affymetrix.com. In each array we assessed whether the printed transcripts were structurally linked to any gene, EST or protein. Gene Ontology (GO) was used as criteria for initial assessment of functional annotation. The purpose of this assessment was to determine which whole chicken genome arrays could be used as reference for structural and functional annotation of other arrays or experimental datasets. Affymetrix chicken genome array was the only one that had been comprehensively structurally and functionally annotated and was selected for further improvement.

### Functional annotation

Further assessment and improvement of GO annotation of the Affymetrix chicken array was necessary. The GO annotations associated with the probe sets on Affymetrix chicken array do not show detail information to support the annotation. For example; were experimental evidence codes are shown there is no any literature referenced to support the annotation. For this reason we decided to re-annotate all gene products linked to the probesets on this array, regardless of their original annotations, in order to provide high quality and standard functional information to the array users. We first used *GORetriever *[22] to download chicken GO annotations for all UniProtKB accessions linked to the probesets. Further annotations for linked gene products with RefSeq number and Ensembl gene identifiers were obtained from AgBase-community databases and Gene Ontology Annotation (GOA) project using an in-house Perl script (*GOMapper.pl*). Additional GO was retrieved by implementing an in-house tool (*ISO.pl*) to transfer the experimental GO annotations from 1:1 chicken-human/mouse/rat orthologs to the corresponding chicken proteins orthologs. The improved GO annotations will be made available publicly via AgBase.

### Additional functional information

In addition to the molecular function, biological process and cellular component annotations provided via the GO, other functional information is also useful for researchers wishing to assess the type of biological information represented by transcript printed on an array. For example, researchers will also benefit by knowing which genes on the array are associated with disease and disorders and which are known drug targets. We used Ingenuity Pathways Analysis (IPA) software to determine known drug targets and significant disease and disorders. The Fischer's exact test was used to calculate a P-value determining the probability that the biological functions, diseases or disorders assigned to the array datasets was due to chance alone.

### Assessment of GO annotation quality (GAQ)

To assess the improvement made in the re-annotated functional annotations of the Affymetrix chicken array, the *meanGAQ *score for GO initially associated with the array was calculated as previously described [20] and compared to that calculated for GO after re-annotations. Briefly, the GAQ score takes into account the quality of GO annotations by quantitatively assessing the level of detail provided by the GO annotation and the type of evidence used to make the functional association. Mathematically the ***GAQ ***score of a GO annotation ***(a) ***can be defined as the product of annotation depth in the ontology ***(Dd) ***and the evidence code rank ***(ECR) ***of the annotation, represented as:

When you have a set of gene products ***(S) ***annotated to a number of GO terms ***(A)***, the ***GAQ ***score can be defined as:

In this study we reported the **mean GAQ **score based on number of gene products ***(n) ***that have GO and was calculated as:

### Development of Array GO Mapper (*AGOM*)

*AGOM *was developed to GO annotate chicken arrays and chicken microarray experimental datasets using improved Affymetrix GO annotations generated in the work described here. The tool is written in Perl and works on both windows and Linux platforms. It requires a tab delimited input file containing the microarray dataset cross references for which the GO annotations are searched. The Affymetrix improved GO data file was used as a database to search from. This database contains 6 cross-reference identifier types, which facilitate mapping between arrays and experimental datasets. *AGOM *works with any type of array (whole genome and specific array platform) and experimental datasets with common identifier(s) between the arrays/datasets and the Affymetrix data. The gene associations are presented in 16 columns according to GO standards (Additional file 3). The depth of a GO term, evidence code rank and GAQ score of individual GO term associated with the Affymetrix GO data are in the last 3 columns of file.

We demonstrated *AGOM *implementation by searching GO annotation for Arizona chicken array (GPL6049) from improved Affymetrix chicken array GO data. The Arizona chicken array was chosen because it has no existing GO associated with its gene products (Table 1). In addition, the Arizona array probes are linked to a variety of identifiers (GenBank accession, Entrez Gene ID and Ensembl ID) that can be used to search the Affymetrix GO data while most of other arrays contain only GenBank accessions (Additional file 3). For example, in this study GenBank accession, Entrez Gene ID and Ensembl ID linked to Arizona array were searched against the improved Affymetrix GO annotations to retrieve corresponding GO records. The output generated from the search includes Arizona array identifiers in the first 5 columns; Oligo_ID (unique ID), GenBank accession, Entrez Gene ID, Ensembl ID and array Spot number. When a match is found the corresponding GO information is added to a tab-delimited output file.

*AGOM *is available via AgBase (http://www.agbase.msstate.edu/; see under Array annotation) where users can use the tool directly online or can download it as a standalone program. When implementing the tool online, users will be given options to retrieve any data associated with the Affymetrix chicken array (Additional file 3). The script is also available upon request and advice is available by e-mail.

## Competing interests

The authors declare that they have no competing interests.

## Authors' contributions

TJB is responsible for the study designing, data generation, data analysis, formulation of workflow for *AGOM *and writing the draft of the manuscript. RK wrote the script for *AGOM *and contributed in manuscript preparation. CRG modified the script, developed *AGOM *webpage and contributed in manuscript preparation. Both FMM and SCB contributed in manuscript preparation and in technical advice. All authors read and approved the final manuscript.

## Supplementary Material

Additional file 1 **Initial assessment of annotation of Affymetrix chicken genome array**. Additional file descriptions text (including details of how to view the file, if it is in a nonstandard format). Click here for file

Additional file 2 **Example of output generated by the Array GO Mapper (AGOM)**. Unique identifier for the Arizona array (Oligo_ID) is displayed in column 1. Column 2–4 displays GenBank accessions, Entrez gene ID and Ensembl gene ID used for mapping. The array spot number is in column 5. The name of database and the corresponding gene product in Affymetrix annotations are shown in column 6 & 7. The GO and name of the GO term are displayed in column 8 & 9 with the evidence code for the annotation in column 10. Column 11 shows the aspects of gene ontology either molecular function (F), cellular component (C) or biological process (P). The GO Annotation Quality (GAQ) score for individual GO term is displayed in column 12 and the date the output was generated in column 13. Click here for file

Additional file 3 **Chicken array platform cross-reference**. Each column represents one array platform showing the identifiers that can be used to search GO annotations from Affymetrix GO data. (+) indicates presence of identifier in the corresponding array platform. (-) indicates absence of identifier in the corresponding array platform. Click here for file
