# Supplementary material for: Facilitating functional annotation of chicken microarray data
Source: BMC Bioinformatics. 2009 Oct 8;10(Suppl 11):S2. doi: 10.1186/1471-2105-10-S11-S2 (PMC3226191; doi:10.1186/1471-2105-10-S11-S2)
Supplement: Additional file 1 — Initial assessment of annotation of Affymetrix chicken genome array. Additional file descriptions text (including details of how to view the file, if it is in a nonstandard format). [file 1471-2105-10-S11-S2-S1.pdf]

Additional file 1. Initial assessment of annotation of Affymetrix chicken genome array

Data	Chicken	Viruses
Total number of Probesets	37,703	689
Number of transcripts	32,774	684
Number of Probesets mapped to gene or gene product	27,852	4
Number of probesets with GO annotation	19,272	3
Number of annotated gene products	12,457	2
Number of annotations	60,621	13
IEA annotation (%)	96	54

IEA: Code for “Inferred from Electronic Annotation”.
